# Prediction of Preterm Delivery from Unbalanced EHG Database

**DOI:** 10.3390/s22041507

**Published:** 2022-02-15

**Authors:** Somayeh Mohammadi Far, Matin Beiramvand, Mohammad Shahbakhti, Piotr Augustyniak

**Affiliations:** 1 AGH University of Science and Technology, 30059 Krakow, Poland; august@agh.edu.pl; 2 Department of Biomedical Engineering, Dezful Branch, Islamic Azad University, Dezful 313, Iran; matin.beiramvand@gmail.com; 3 Biomedical Engineering Institute, Kaunas University of Technology, 51423 Kaunas, Lithuania; mohammad.shahbakhti@ktu.edu

**Keywords:** preterm labor, prediction, electrohysterogram, empirical mode decomposition, support vector machine

## Abstract

Objective: The early prediction of preterm labor can significantly minimize premature delivery complications for both the mother and infant. The aim of this research is to propose an automatic algorithm for the prediction of preterm labor using a single electrohysterogram (EHG) signal. Method: The proposed method firstly employs empirical mode decomposition (EMD) to split the EHG signal into two intrinsic mode functions (IMFs), then extracts sample entropy (SampEn), the root mean square (RMS), and the mean Teager–Kaiser energy (MTKE) from each IMF to form the feature vector. Finally, the extracted features are fed to a k-nearest neighbors (kNN), support vector machine (SVM), and decision tree (DT) classifiers to predict whether the recorded EHG signal refers to the preterm case. Main results: The studied database consists of 262 term and 38 preterm delivery pregnancies, each with three EHG channels, recorded for 30 min. The SVM with a polynomial kernel achieved the best result, with an average sensitivity of 99.5%, a specificity of 99.7%, and an accuracy of 99.7%. This was followed by DT, with a mean sensitivity of 100%, a specificity of 98.4%, and an accuracy of 98.7%. Significance: The main superiority of the proposed method over the state-of-the-art algorithms that studied the same database is the use of only a single EHG channel without using either synthetic data generation or feature ranking algorithms.

## 1. Introduction

Preterm birth, also referred to as premature delivery, is defined as giving birth sooner than after 37 weeks of gestation. Preterm delivery is considered a complex condition that occurs due to several biological, mental, and clinical factors, such as, but not limited to, ascending infection, hypoxic-ischemic damage to the uteroplacental unit, chronic stress and fetal and/or uterine developmental malformations, maternal stress, depression, multiple gestations, abortion and short cervical lengths, surgery, ethnicity, and lifestyle [1,2]. However, the extent to which these factors are associated with premature delivery has not yet been proven, as almost 50% of preterm births happen without observation of the mentioned factors [3].

According to the World Health Organization (WHO), preterm birth is the leading cause of fetal morbidity and mortality, and it is increasing all around the world. A recent report from the WHO shows that more than 15 million neonates are delivered prematurely, of which 1 million die each year due to complications [4]. Even the survivors are exposed to various lifelong disabilities, such as, but not limited to, learning difficulties and vision or hearing impairments. Regardless of its complications, the price of medical care for preterm babies imposes a significant financial burden on the family and society, as it costs 5- to 10-times more than a term birth [5]. Thus, early prediction of preterm delivery, combined with appropriate medication to prevent this phenomenon, can greatly minimize the corresponding complications for both the mother and the baby, and reduce the economic load on public health systems.

To predict preterm labor, several physiological measurements, such as tocodynamometer [6], ultrasound [7], fetal fibronectin [8], and internal uterine pressure [9] have been studied. Despite adequate performance, the mentioned measurements are either subjective or invasive [10]. Recently, the analysis of electrical representation of uterus contractions collected from the abdominal surface of pregnant women, known as electrohysterography (EHG), has been evidenced as a reliable tool for the prediction of premature delivery, as it is a non-invasive procedure that can be automated to reduce human intervention [11,12,13].

To profile EHG signals for preterm labor prediction, a wide range of temporal and spectral measurements have been investigated [14,15,16]. Khalil and Duchene [17] presented a hybrid algorithm based on the dynamic cumulative sum and multiscale decomposition to detect different events of EHG signals. Moslem et al. [18] investigated the irregularity of EHG signals by sample entropy (SampEn) and showed its suitability for the discrimination of term and preterm pregnancies. Diab et al. [19] examined time reversibility, SampEn, Lyapunov exponents, and delay vector analysis to classify term and preterm EHG signals, and reported that time reversibility gives the best results. Fele-Zorz et al. [20] compared linear and nonlinear features to analyze EHG recordings for the identification of term and preterm deliveries, demonstrating that non-linear ones are more appropriate. Using wavelet package decomposition (WPD), Alamedine et al. [21] examined the adaptability of several spectral features for term–preterm labor classification, and concluded that the mean power frequency gives the best results. Ahmed et al. [22] showed the superiority of multivariate multiscale fuzzy entropy over multivariate multiscale entropy for the classification of term and preterm cases using EHG signals. Horoba et al. [23] studied various temporal and spectral features of EHG signals, and reported the median frequency as the most suitable feature for distinguishing term and preterm records. Yet, the main limitation of the above-mentioned studies is that they consider only one measure for the discrimination of term and preterm cases, compromising the performance of the learning method [24].

## 2. Related Works

One of the most-studied EHG-related delivery databases is Term–Preterm ElectroHysteroGram DataBase (TPEHG DB), which contains 262 term and 38 preterm labor records, each with three channels [20]. A detailed description of the database is provided in Section 4.1. One of the greatest challenges for the aforementioned database is the imbalanced class distribution of term and preterm EHG cases, which was resolved by employing data-generation methods such as the synthetic minority oversampling technique (SMOTE) [25] or the adaptive synthetic sampling approach (ADASYN) [26]. Fergus et al. [27] extracted the peak frequency, median frequency, root mean square (RMS), and SampEn from a single EHG channel band-pass filtered at 0.34–1 Hz, and employed SMOTE to overcome the imbalance problem. The best result was achieved by the polynomial classifier with an average sensitivity of 97%, a specificity of 90%, and an area under the curve (AUC) of 0.95. With features similar to [27] calculated from a single EHG channel band-pass filtered at 0.3–3 Hz plus the min–max oversampling method, Hussain et al. [28] reported 91.2% for sensitivity, 94.5% for specificity, and 92.7% for accuracy with the self-organized network inspired by the immune algorithm (SONIA) classifier. Smrdel et al. [29] used the adaptive autoregressive method to estimate the median frequency and SampEn from a single EHG channel band-pass filtered at 0.3–4 Hz. After employing SMOTE, quadratic discriminant analysis and a support vector machine showed accuracies of 86% and 87%, respectively. In another study, Fergus et al. [30] extracted SampEn, waveform length, log detector, and variance from a single EHG channel similar to [27]. Using SMOTE for the data balancing, the authors reported an AUC of 0.94, a sensitivity of 91%, and a specificity of 84%, achieved by mixture of a Levenberg–Marquardt-trained feed-forward neural network, a radial basis function neural network, and random neural network classifiers. Peng et al. [31] investigated the appropriateness of 31 temporal and spectral features extracted from three EHG channels, which were collected before the 26th week of gestation. After selecting 15 features and employing ADASYN, a random forest classifier showed an accuracy of 93%, a sensitivity of 89%, and a specificity of 97%. Degbedzui et al. [32] employed autoregressive modeling to extract a new measure, called centroid frequency estimation, from a single EHG channel. After employing ADASYN, an accuracy of 99.72%, a specificity of 99.96%, and a sensitivity of 99.48% was achieved by the SVM classifier. Ye-Lin et al. [33] extracted 203 temporal, spectral, and nonlinear features from 326 multichannel EHG recordings and employed SMOTE to balance the database. After feature selection by a genetic algorithm, the authors reported a mean F1 score of 92.04% using an ensemble classifier. Later on, Ye-Lin et al. [5] showed the efficiency of entropy measures for the classification of the term–preterm EHG recordings by a linear discriminant analysis (LDA) classifier with an average F1 score of 90.1%. Yang et al. [34] extracted RMS, median frequency, peak frequency, and sample entropy from the EHG signals, and applied SMOTE for overcoming the data imbalance issue. After employing five classifiers, the authors reported the best accuracy, of 85%, by the gradient boosting classifier (GBC). Xu et al. [35] presented a network theory-based algorithm for the prediction of preterm labor. Using a partition-synthesis method for overcoming the data balancing and SVM for classification, an accuracy of 91% was achieved.

As EHG signals are generated from a huge amount of intricately interconnected cells, uterus contractions are expected to display strong non-stationarity [20]. Consequently, employing non-stationary algorithms for feature extraction was expected to improve the classification results. The most prominent example of such methods is empirical mode decomposition (EMD) [36], which splits the input signal into several frequency components (ordered from high to low), called intrinsic mode functions (IMFs), without requiring any prior knowledge such as basis functions in the wavelet transform. For this reason, some researchers performed EMD on EHG data, then extracted features from the decomposed components. Ren et al. [37] used the Shannon entropy of the first ten decomposed IMFs to form the feature vector. After applying SMOTE, several classifiers were used to discriminate term and preterm deliveries. According to the authors, the best result was achieved based on the Adaboost classifier with an AUC of 0.986. N. Sadi et al. [38] used a balanced data set of EHG records (26 term and 26 preterm records) and extracted seven features from the IMF3 and IMF6 of two EHG channels. After employing a linear SVM classifier, an average accuracy of 95.70%, a sensitivity of 98.40%, a specificity of 93%, and an AUC of 0.95 were achieved. Acharya et al. [39] employed EMD to extract 11 IMFs from EHG records that were subsequently decomposed by WPD to 6 levels. After ranking significant features and employing ADASYN, SVM achieved an accuracy of 96.25%, a sensitivity of 95.08% and a specificity of 97.33%. Khan et al. [40] extracted nine features from the second to fifth IMFs. After using ADASYN for data balancing and SVM for classification, an accuracy of 98% was obtained.

While the mentioned investigations showed satisfactory results for the prediction of preterm labor, the majority of them employed synthetic data generation to overcome the imbalance problem, which may cause misleading results [11,29,30], i.e., if an algorithm detects preterm labor cases with a sensitivity of 90% after data balancing, it is not clear how much of that 10% of misclassified preterm cases are related to real data. Indeed, if such a misclassification is strongly related to the real data, not the synthetically genereated ones, the performance may not be reliable. To this aim, this research proposes an automatic algorithm to predict preterm labor from a single EHG channel without employing any synthetic data-generation algorithms. Our hypothesis is that EHG records related to preterm labor contain stronger contractions than term ones; therefore, instead of extracting many features, we consider only three features that properly address such differences. In addition, given our hypothesis—that EHG signals related to preterm labor cases have stronger contractions—feature extraction is better to be performed on EHG signals decomposed by EMD, where the EHG signal is represented by several components with different frequency ranges. After the feature extraction, k-nearest neighbors (kNN), SVM, and decision tree (DT) classifiers are employed to investigate the effectiveness of the proposed features.

## 3. Methods

### 3.1. The Proposed Method

**Figure 1:**
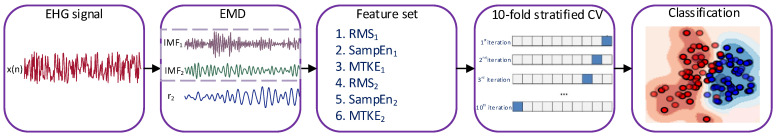
The block diagram for the discrimination of the deliveries.

The block diagram of the proposed method is shown in Figure 1. In short, the following procedures will be employed: (i) two IMFs are extracted from EHG signals; (ii) three features are extracted from these IMFs to form the feature vector; (iii) the extracted features are fed to three classifiers for studying the best possible discrimination of the term and preterm cases. The following subsections explain the proposed method in detail.

### 3.2. Empirical Mode Decomposition

The basis of EMD is to decompose the input signal x(n) into *m* number of intrinsic mode functions (IMFs) and one residual signal r(n), where the original signal can be reconstructed as follows:

(1) $$x\left(n\right)=\sum_{j=1}^{m}IMF_{j}+r_{m}\left(n\right).$$

Indeed, EMD decomposes the input signal from high to low frequency components as the level of decomposition, *j*, increases. Since the input signal is decomposed in the time domain, and the length of the decomposed components and the original signal is equal, EMD preserves the characteristics of varying frequencies [36]. An IMF is defined as a function that satisfies the two conditions:

1. In the whole data set, the number of extrema and zero-crossings must either be equal or differ at most by one;
2. At any point, the mean value of the envelope defined by the local maxima and the envelope defined by the local minima is zero.

The second condition is ideal and may be not achieved in practice; therefore, it is controlled by a threshold. If the mean value of the lower and upper envelopes is below 0.2, it is considered an IMF. The process of IMF extraction from x(n), known as the sifting process, is described as follows:

1. Extract the local minima and local maxima from x(n);
2. Create the upper and lower signal’s envelopes using cubic spline;
3. Compute the local mean signal, $m_{1}\left(n\right)$, by averaging the upper and lower signal’s envelopes;
4. Subtract $m_{1}\left(n\right)$ from x(n) to obtain the first possible IMF candidate $y_{1}\left(n\right)=x\left(n\right)-m_{1}\left(n\right)$.

Now, it should be investigated whether $y_{1}\left(n\right)$ fulfills IMF’s conditions. If not, $y_{1}\left(n\right)$ is considered as a new signal, and steps 1–4 have to be repeated. This process is continued *k* times until $y_{1k}\left(n\right)$ is chosen as the first IMF. In order to reach a series of IMFs, the residue, $r_{1}\left(n\right)$, should be generated as follows:

(2) $$r_{1}\left(n\right)=x\left(n\right)-IMF_{1}.$$

Then, the sifting process is performed on $r_{1}\left(n\right)$ to obtain the second IMF.

Considering the fact that EMD decomposes a signal from high- to low-frequency components, and that our hypothesis that EHG signals related to preterm labor cases might represent stronger contractions, i.e., show higher frequency components, we employ only the first two IMFs. In order to decompose EHG signals by EMD, each signal was windowed into 1-min segments, and the first two IMFs were extracted.

### 3.3. Feature Extraction

The first step for the true segmentation of term and preterm deliveries is to select distinctive features. Our presumption is that preterm EHG signals contain stronger contractions than term ones; thus, features able to represent this property should be extracted. In this paper, we extracted three features, RMS, SampEn [41], and mean Teager–Kaiser energy (MTKE) [42], from the first two IMFs. Although the adequacy of the employed features has been proven when directly extracted from EHG signals, better performance might be achieved if they are extracted from decomposed EHG signals, where high-frequency components, i.e., the first two IMFs, are the only ones considered. The motivation behind using these three features is their capability to distinguish stronger contractions, as EHG signals related to preterm cases are expected on the physiological background to show such behavior. Indeed, stronger contractions show higher amplitude (RMS), uncertainty (entropy), and energy (MTKE).

RMS is defined as the square root of the arithmetic mean of the squares of the values, expressed as follows:

(3) $$\mathrm{RMS}=\sqrt{\frac{1}{N}\sum_{n=1}^{N}x^{2}\left(n\right)}.$$

As our assumption is that preterm EHG records contain stronger contractions, it is expected that the RMS of term and preterm records show a meaningful difference [14].

SampEn is a modification of approximation entropy without independence from the data length. For a given signal with a length of *n*, it can be expressed as the negative logarithm of conditional probability that two sequences are similar for *m* point within a tolerance value *r*, excluding any self-matches. Thus, it can be represented as:

(4) $$\mathrm{SampEn}\left(m,r,n\right)=-\ln\left(\frac{A}{B}\right),$$

where $A=\frac{\left(n-m-1)(n-m\right)}{2}A^{m}\left(r\right)$ and $B=\frac{\left(n-m-1)(n-m\right)}{2}B^{m}\left(r\right)$. $A^{m}\left(r\right)$ and $B^{m}\left(r\right)$ stand for the probabilities of two sequence matches for m+1 and *m* points, respectively. SampEn represents the irregularity of the signal. As stronger contractions can also increase irregularity, it can be expected as a proper feature for the term and preterm segmentation [5,18]. SampEn requires two parameters to be adjusted before implementation, embedding dimension *m* and scaling factor *r*. In this paper, we used m=3 and r=0.15, as suggested in [5].

TKE is a well-known tool for the detection of muscle-contraction onsets from electromyogram signals. In this paper, we use the mean of TKE as follows:

(5) $$\mathrm{MTKE}=\mu (x^{2}\left(n\right)-x\left(n-1\right)x\left(n+1\right)),$$

where μ represents the mean. The MTKE calculates the energy of a signal based on its amplitude and frequency content; hence, its higher average value can represent preterm cases [39].

As was already mentioned, each EHG signal is decomposed into two IMFs; therefore, 6 features from each channel are extracted. It should be noted that, after segmenting EHG signals into 1-min windows and the IMF extraction, each feature was computed from all those windows, and the average of them was considered as the final feature. In order to normalize the features’ scale, each feature column is subtracted from its mean and divided by its standard deviation.

### 3.4. Classifiers

Three classifiers were learned and tested to best distinguish the term and preterm delivery signs based on the above-presented features.

#### 3.4.1. k-Nearest Neighbors

kNN is a simple supervised machine-learning algorithm, widely employed for classification and regression problems. The basis of kNN is to separate data points by using a distance function. Indeed, kNN performs the classification by the majority vote of neighbors, where each data point is attributed with a label that has the closest neighbors. Therefore, kNN presumes that resembling data are in close proximity. There are two parameters that influence the classification results of kNN: the number of neighbors *K* and the distance metric. The determination of both parameters is an experimental task [43].

#### 3.4.2. Support Vector Machine

SVM is one the most efficient supervised machine-learning algorithms that have been extensively used in dual classification problems. The main advantage of SVM is its capability to separate and handle multiple continuous and categorical variables. In general, SVM generates a hyperplane in multi-dimensional space to distinguish different classes. Compared to other classifiers, SVM’s kernel-selection property provides a better solution to deal with complex data. Yet, the optimization of kernel parameters is a time-consuming procedure [44].

#### 3.4.3. Decision Tree

The decision tree deals with the classification problem as a form of tree structures. It decomposes the database into smaller subsets that are incrementally developed. As the final results, decision and leaf nodes will be given, where decision nodes have two or more branches and leaf nodes represent the classification results [45]. Compared to the SVM, the classification result of DT depends on more required parameters in order to be tuned accurately. As a consequence, DT can be more operator-dependent.

## 4. Evaluation

### 4.1. Data

**Figure 2:**
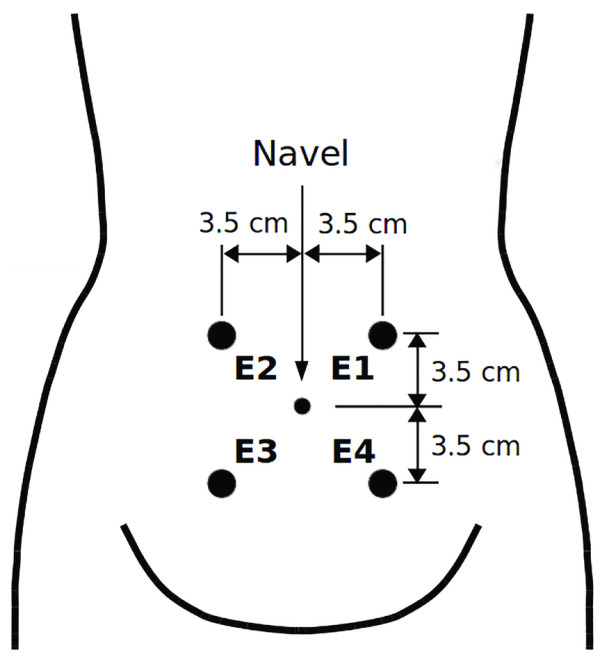
The placement of EHG electrodes, adopted from [46].

The EHG records included in the TPEHG DB database were collected from 1997 to 2005 at the University Medical Centre Ljubljana, Department of Obstetrics and Gynecology [20]. The TPEHG DB contains 300 EHG records, of which 262 records were of term and 38 records were of preterm deliveries. According to pregnancy weeks, these records are categorized into two groups, where 143 term and 19 preterm records were collected before the 26th week of gestation, and 119 term and 19 preterm records were collected during or after the 26th week of the gestation. Each record is comprised of three channels, recorded from four electrodes placed on the abdominal surface of pregnant women, as shown in Figure 2.

**Figure 3:**
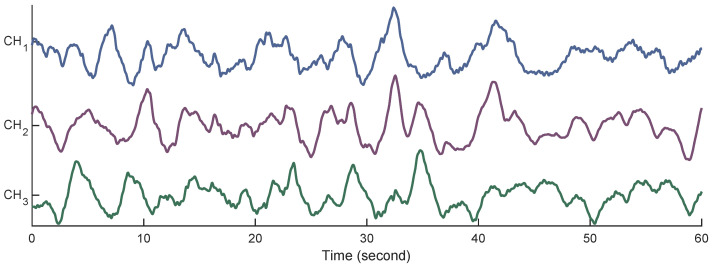
Examples of the EHG signals from all three channels.

Using differences in the electrical potentials of the electrodes, three channels were produced as $CH_{1}=E2-E1$, $CH_{2}=E2-E3$, and $CH_{3}=E4-E3$. Each record lasted for 30 min and was sampled at 20 Hz with 16-bit resolution over a range of ±2.5 millivolts. After data collection, a fourth-order Butterworth band pass filter with cut-off frequencies of 0.08 and 4 Hz was employed to filter the raw EHG signals. To mitigate the transient effect of filtering, the first and last 180 s of each record were removed [20]. Figure 3 shows 1 min of the EHG signals from all three channels after filtering.

### 4.2. Imbalanced Database Issue

The major problem of the TPEHG DB database is the imbalance of EHG data for term and preterm classes, as only 13% of data corresponds to preterm cases. In this situation, the classifiers may be biased against the preterm labor cases [47]. Indeed, by using the simple k-fold cross-validation, it is possible that few folds do not have preterm cases. The most straightforward strategy addressed in the literature is to generate synthetic preterm features by algorithms such as SMOTE or ADASYN. Yet, some studies argued that such a strategy may lead to misleading results [11,29,30]. To overcome this issue, we employed stratified k-fold cross-validation, which randomly splits the database into *k* subsets and guarantees the existence of both classes in all subsets.

### 4.3. Evaluation Metrics

In order to evaluate the performance of the classifiers, the sensitivity (*Se*), specificity (*Sp*), and accuracy (*Acc*) are computed as follows:

(6) $$Se=\frac{TP}{TP+FN}\times 100,$$

(7) $$Sp=\frac{TN}{TN+FP}\times 100,$$

(8) $$Acc=\frac{TP+TN}{TP+TN+FP+FN}\times 100,$$

where TP and FN represent the number of correctly and wrongly classified preterm cases, and TN and FP stand for the number of correctly and wrongly classified term cases. To validate the performance of the classifiers, a 10-fold stratified cross-validation with 30 repetitions was performed. After splitting the data into 10 subsets, the training and testing procedures are performed in such a way that, each time, 9 subsets are used for training and 1 subset is used for testing. Consequently, the classification results are taken as the average of 10 repetitions for training and testing. It should be noted that during these 10 repetitions, the obtained testing results are independent of the previously trained classifier. After finding the best results for each classifier, the AUC is computed as follows:

(9) $$\mathrm{AUC}=\int Se\left(T\right){\left(1-Sp\right)}^{\prime }\left(T\right)dT,$$

where *T* is the threshold related to the binary classifier.

## 5. Results and Discussion

As mentioned in the state-of-art review, each classifier requires the accurate setting of parameters prior to performing the experiment. In this paper, we report the performance of each classifier with an alternation of the most-prominent parameter. The remaining parameters are set as the default. Compared to the state-of-art methods, we show the employed features better discriminate term and preterm cases, supporting our claim with quantitative results. Furthermore, the main superiority of the proposed method over the state-of-the-art algorithms is to employ only real EHG signals. The experiments were implemented in a MATLAB 2019 environment using a personal computer with a 3.2 GHz core i7 CPU and 8-GB memory.

**Figure 4:**
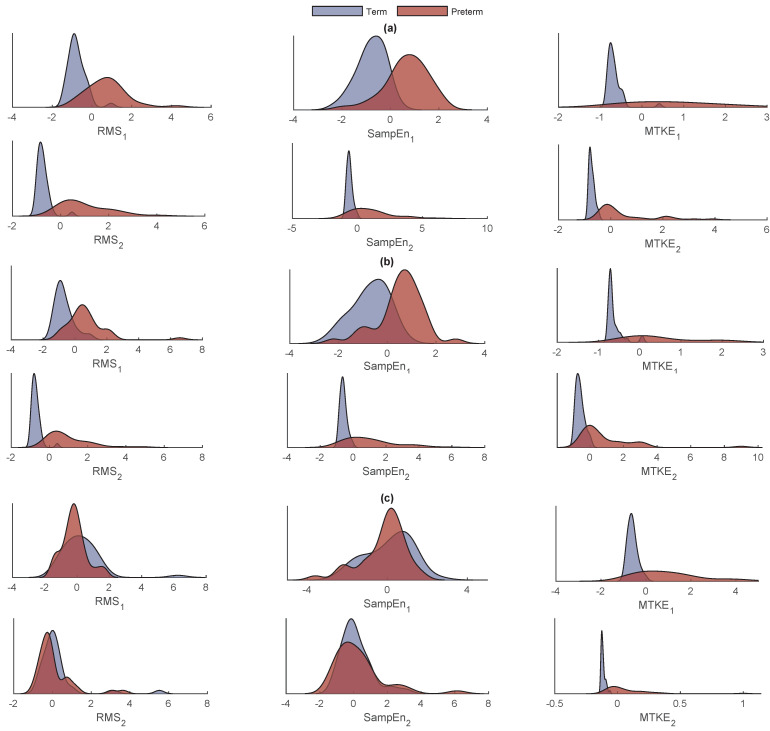
The distribution of the extracted features from IMF1 (first row) and IMF2 (second row) from (**a**) $CH_{1}$, (**b**) $CH_{2}$, and (**c**) $CH_{3}$.

Figure 4 shows the distribution of the extracted features. As for kNN, the distance metric and the number of neighbors should be specified first. According to the literature, the most common distance metric for kNN classifiers is Euclidean; thus, the number of neighbors plays the most important role. For this aim, a different number of neighbors, i.e., 2, 4, 8, 10, and 12, are examined. As shown in Table 1, the best results were achieved from $CH_{2}$ by 4 neighbors with a mean Se of 86.9%, Sp of 98.0%, and Acc of 96.6%, followed by 4 neighbors with a mean Se of 86.1%, Sp of 97.8%, and Acc of 96.3% from $CH_{1}$.

As for SVM, linear, radial basis function (RBF), and polynomial (Poly) kernels were used. It should be noted that kernel parameters that maximize the margin between term and preterm cases and minimize the misclassification rate were adjusted automatically in a MATLAB 2019 environment. Table 2 shows the classification results by SVM. As displayed, the best results were reached through $CH_{1}$ features using a poly kernel with an average Se of 99.5%, Sp of 99.7%, and Acc of 99.7%, followed by a poly kernel with an average Se of 93.6%, Sp of 99.6%, and Acc of 98.9% from $CH_{2}$.

Regarding the decision tree classifier, there are two parameters that can influence the performance: the maximum number of split (MNS) and the minimum leaf size (MLS). As for MNS, we optimized the DT classifier in the MATLAB environment and a MNS of 6 was considered as the optimal value. Thus, we investigated different numbers of leaves, i.e., 10, 20, 30, 40, and 50. Table 3 displays the classification results using DT. As can be observed, the best result was obtained by 20 leaves from $CH_{1}$, with a mean Se of 100%, Sp of 98.4%, and Acc of 98.7%, followed by 30 leaves, with an average Se of 100%, Sp of 97.7%, and Acc of 98.2%. Compared to other two classifiers, the results obtained by DT seem to be more robust to the different channels.

**Figure 5:**
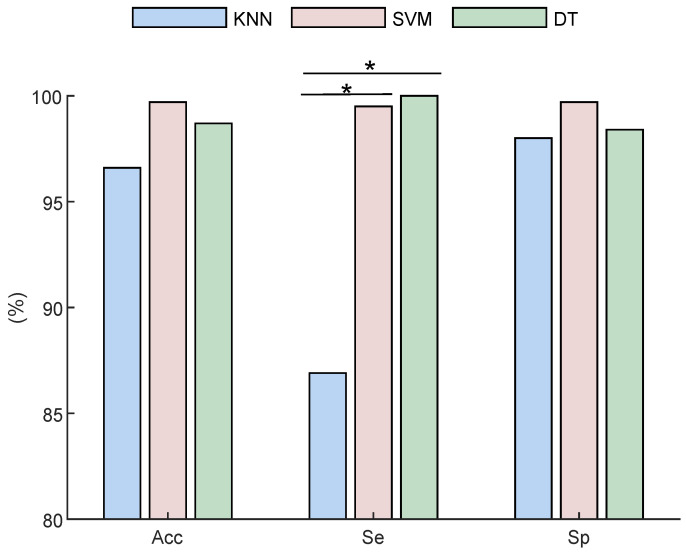
The performance comparison of all three classifiers. * stands for *p* < 0.05.

According to Table 1, Table 2 and Table 3, the best performance results are obtained by SVM and DT classifiers using extracted features from $CH_{1}$. Figure 5 compares the best obtained results in terms of Se, Sp, and Acc by all classifiers. As displayed, there is only a significant difference (p<0.05) between Se values of kNN vs. SVM and DT.

**Figure 6:**
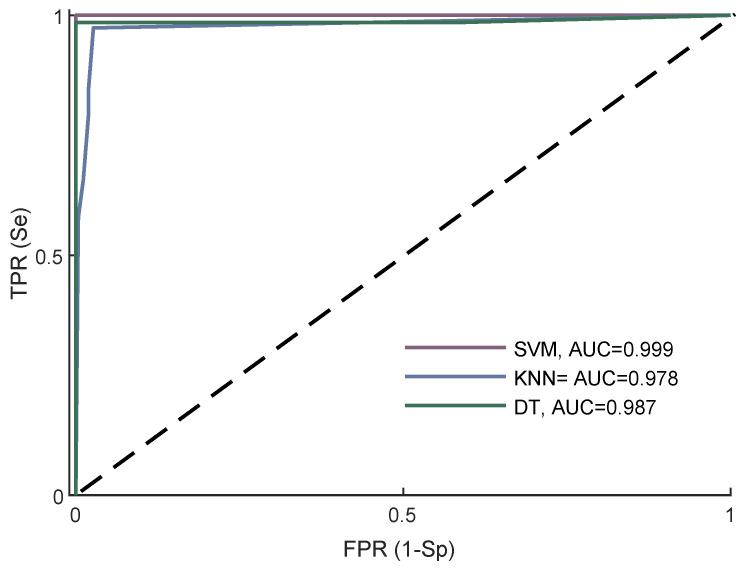
ROC of all classifiers with their best performance.

The receiver operating characteristic (ROC) curves of all classifiers are shown in Figure 6. As can be observed, SVM achieved the highest AUC of 0.999, followed by DT and kNN, with 0.987 and 0.978, respectively.

Table 4 compares our algorithm with state-of-the-art algorithms tested against the TPEHG DB database. The most noticeable advantage of our study is the use of original EHG records without synthetic data generation. In addition, our algorithm does not require feature ranking procedures. Instead, we employed three physiology-justified features which could properly discriminate term and preterm cases. Considering the similarity of feature extraction to our study, the best obtained results based on EMD analysis were achieved by the [39], with Se of 95.08%, Sp of 97.32%, and Acc of 96.25. On the other hand, the resemblance of our results with [32] shows the applicability of the extracted features even when imbalanced EHG records were employed. Regardless the obtained results, due to low wearable complexity, i.e., single EHG channel, and computational cost, the proposed method could be integrated for using in home-based surveillance in indoor environments. Indeed, single-lead EHG monitoring paves the way for multimodal pregnancy monitoring in home-care scenarios, which is increasingly stressed in countries with the lowest population growth rates. The use of a single channel opens the possibility of selecting the best channel out of the three used in regular monitoring and of modifying the selection with changes of environmental conditions or the mother’s position, which optimizes the monitoring quality-to-cost ratio and the accessibility of the method. Alternatively, a single-channel EHG record may be considered as a part of a complex well-being record (including mother motion and fetus heart rate measurements) that documents the course of pregnancy while searching for possible threats.

## 6. Conclusions and Future Works

This paper presents an automatic algorithm for the accurate classification of term and preterm deliveries using a single EHG channel. The obtained results confirmed the adequacy of extracted features as no synthetic data-generation or feature-ranking algorithms were necessary. Indeed, our results suggested that employing features that properly characterize the contractions can avoid such extra processing. In future works, we should investigate (i) employing the categorical characteristics of each subject as the complementary features in addition to the proposed ones, (ii) the performance of employed features on other versions of filtered EHG signals with different cut-off frequencies (e.g., 0.3–1 Hz), (iii) the classification of EHG data based on the recorded weeks to investigate how far ahead preterm labor can be predicted, and (iv) the consistency of classification with the shortening of records.

## Figures and Tables

**Table 1:** kNN performance for different channel configurations. The best obtained results are in bold.

No. of K	Channel	Se	Sp	Acc
2	CH1	81.2 %	95.1 %	93.9 %
	CH2	78.9 %	94.0 %	91.8 %
	CH3	77.3 %	95.4 %	93.2 %
4	CH1	86.1 %	97.8 %	96.3 %
	CH2	**86.9 ** %	**98.0** %	**96.6** %
	CH3	82.9 %	97.6 %	96.7 %
8	CH1	82.1 %	98.7 %	96.6 %
	CH2	80.0 %	98.0 %	95.8 %
	CH3	81.3 %	99.6 %	97.2 %
10	CH1	79.2 %	98.8 %	96.2 %
	CH2	79.0 %	98.3 %	95.9 %
	CH3	80.0 %	99.6 %	97.1 %
12	CH1	77.1 %	98.9 %	96.0 %
	CH2	76.7 %	98.2 %	95.4 %
	CH3	78.0 %	99.6 %	96.8 %

**Table 2:** SVM performance for different channel configurations. The best results are in bold.

Kernel	Channel	Se	Sp	Acc
Linear	CH1	77.5 %	98.4 %	95.8 %
	CH2	78.9 %	98.5 %	96.0 %
	CH3	85.0 %	99.6 %	97.7 %
RBF	CH1	82.8 %	98.8 %	96.7 %
	CH2	83.7 %	98.3 %	96.4 %
	CH3	76.8 %	100 %	97.0 %
Poly	CH1	**99.5** %	**99.7** %	**99.7** %
	CH2	93.6 %	99.6 %	98.9 %
	CH3	88.4 %	99.0 %	97.7 %

**Table 3:** Decision tree performance for different channel configurations. The best results are in bold.

MLS	Channel	Se	Sp	Acc
10	CH1	93.4 %	96.4 %	96.1 %
	CH2	87.8 %	92.8 %	92.1 %
	CH3	89.7 %	94.8 %	94.5 %
20	CH1	**100** %	**98.4** %	**98.7** %
	CH2	91.0 %	96.9 %	96.2 %
	CH3	91.7 %	96.8 %	96.2 %
30	CH1	100%	97.7 %	98.2 %
	CH2	94.1%	96.7 %	96.2 %
	CH3	91%	97.0 %	96.2 %
40	CH1	97.2 %	96.8 %	96.8 %
	CH2	97.1 %	96.8 %	96.9 %
	CH3	100 %	97.3 %	97.6 %
50	CH1	100%	93.2 %	94.1 %
	CH2	100%	93.3 %	94.1 %
	CH3	100%	93.2 %	94.0 %

**Table 4:** The comparison of our study with sate-of-the-art algorithms. * means feature selection was performed before the classification.

blackWork	Data Balancing	Channel	No. Data	Classifier	Acc	Se	Sp	AUC
black [27]	Yes (SMOTE)	CH3	262 term; 38 preterm	polynomial	–	96%	90%	0.95
[28]	Yes (Min–Max)	CH3	150 term; 19 preterm	SONIA	92.7%	91.2%	94.5%	0.93
[29]	Yes (SMOTE)	CH3	262 term; 38 preterm	SVM	87%	96%	79%	–
[30]	Yes (SMOTE)	CH3	262 term; 38 preterm	Combined *	–	91%	84%	0.94
[31]	Yes (ADASYN)	CH1-3	143 term; 19 preterm	RF *	93%	89%	97%	0.962
[32]	Yes (ADASYN)	CH1	262 term; 38 preterm	SVM	99.72%	99.48%	99.96%	–
[37]	Yes (SMOTE)	CH3	262 term; 38 preterm	Adaboost	–	–	–	0.986
[38]	No	CH1-2	26 term; 26 preterm	SVM *	95.70%	98.40%	93%	0.95
[39]	Yes (ADASYN)	CH1-3	262 term; 38 preterm	SVM *	96.25%	95.08%	97.33%	–
[33]	Yes (SMOTE)	CH1-3	275 term; 51 preterm	Ensemble *	91.64%	96.23%	87.04%	98.13
[5]	Yes (SMOTE)	CH1-3	275 term; 51 preterm	LDA *	89.2%	98.4%	79.9%	0.936
[34]	Yes (SMOTE)	CH1-3	262 term; 38 preterm	GBC	85%	-	-	0.91
[35]	Yes (Partition-Synthesis)	CH1-3	275 term; 51 preterm	SVM *	91%	89.0%	93%	0.97
[40]	Yes (ADASYN)	CH1-3	262 term; 38 preterm	SVM *	98.5%	98.4%	98.4%	-
Ours	No	CH1	262 term; 38 preterm	SVM	99.7%	99.5%	99.7%	0.999

## Data Availability

This database is publicly available at https://physionet.org/content/tpehgdb/1.0.1/, accessed on 20 October 2020.

## Abbreviations

The following abbreviations are used in this manuscript:

EHG	electrohysterogram
EMD	empirical mode decomposition
IMF	intrinsic mode functions
SampEn	sample entropy
RMS	root mean square
MTKE	mean Teager–Kaiser energy
kNN	k-nearest neighbors
SVM	support vector machine
LDA	linear discriminant analysis
Acc	accuracy
Se	sensitivity
Sp	specificity
AUC	area under the curve
SMOTE	synthetic minority oversampling technique
ADASYN	adaptive synthetic sampling approach
GBC	gradient boosting classifier
